# A web-based tool for rapid and accurate craniometric differentiation of clouded leopard species

**DOI:** 10.1038/s41598-025-96080-w

**Published:** 2025-04-02

**Authors:** Chrishen R. Gomez, Andrew C. Kitchener, Andrew J. Hearn, Ibnu Maryanto, Paul J. Johnson, David W. Macdonald, Nobuyuki Yamaguchi

**Affiliations:** 1 Wildlife Conservation Research Unit, Department of Biology, The Racanati-Kaplan Centre, University of Oxford, Oxford, UK; 2 Department of Natural Sciences, National Museums Scotland, Chambers Street, Edinburgh, EH1 1JF UK; 3 School of Geosciences, Drummond Street, Edinburgh, EH8 9XP UK; 4 Museum Zoologicum Bogoriense Center for Research on Biosystematica and Evolution-BRIN, Indonesian Institute of Sciences (LIPI), Jl. Raya Cibinong KM 47, Cibinong, Indonesia; 5 Institute of Tropical Biodiversity and Sustainable Development, University of Malaysia Terengganu, 21030 Kuala Nerus, Terengganu Malaysia

**Keywords:** Wildlife-trade, Craniometrics, Clouded leopard, Morphology, Museum, Southeast Asia, Zoology, Taxonomy, Software

## Abstract

The illegal wildlife markets of Southeast Asia are bolstered by organised criminal networks and the region’s rich density of charismatic wildlife. Forensic tools identifying species and their origins are vital to combat wildlife crime. However, many require expensive technology and skilled personnel, limiting their use in rural trade hotspots. This study introduces a replicable statistical framework, using skull morphometrics, to distinguish related species with simple measurements. We developed a web-based classifier trained on clouded leopard (*Neofelis* spp.) skulls from museum collections across Europe, Asia and the U.S.A., a genus often targeted in wildlife trade. Our categorical predictive model, based on two key metrics, the fronto-nasal “pit” and m1 talonid morphology achieved 97% accuracy (*p* < 0.005). A continuous predictor model, using postorbital width, achieved 80.6% accuracy for males and 85.6% for females (both *p* < 0.05). These models were encoded into a free, user-friendly web app, enabling practitioners in remote areas to distinguish these two species easily. This tool not only supports anti-trafficking efforts but also enables museum curators to correctly assign provenance to clouded leopard skulls with uncertain origins.

**Supplementary Information:**

The online version contains supplementary material available at 10.1038/s41598-025-96080-w.

## Introduction

Wildlife forensics is a crucial field devoted to the science of identifying species and their origin using a wide array of technological tools^1^. Forensic tools are an important first line of defence in the globalised world of trade, where illegal and legal wildlife trade is projected to grow in tandem with international socioeconomic inequality^2^. Large charismatic predators are often a key target of illegal wildlife trade, which can drive local extinction trends in wild populations of predators^3^. Skulls are a popular item of trade as an embodiment of memory for many cultures spanning entire civilisations^4^. For large felids, distinguishing species in the field (e.g., wildlife markets), using skull morphometrics, can be a difficult task due to both the close relationships and convergent evolutionary adaptations that have made them morphologically similar^5,6^. This is problematic for regulating the trade of wildlife specimens and their parts. While it is possible to distinguish species using high-dimensional skull morphometrics^7^ or genetic markers^8^, the sheer volume of traded skulls needing classification is large and would benefit from a simpler species diagnostic tool.

Clouded leopards (*Neofelis* spp.) are the smallest of the “big cats” within the Pantherinae. Their adaptations and size are consistent with a semi-arboreal life in the dense canopies of tropical rainforests in Southeast Asia. Clouded leopards have the largest canines for their skull size amongst the extant felids, and hence, are often referred to as an example of convergence with the extinct sabre-toothed cats, although they do not necessarily show the craniomandibular shape similar to that of the sabre-tooths with extremely large canines^9,10^. Due primarily to their large canines relative to skull size, clouded leopards are exceptions in terms of the craniofacial evolutionary allometry amongst the extant felids^9,11,12^. Clouded leopards also have a unique taxonomic history due to their relatively recent reclassification into two distinct species after morphological and genetic analysis^6,7,13^, the mainland clouded leopard (*Neofelis* *nebulosa*) and Sunda clouded leopard (*Neofelis* *diardi*). The close evolutionary history of both *Neofelis* species makes them ideal to study the utility of simple craniometric models for classifying species.

The recent taxonomic revisions of clouded leopards have also precipitated a detailed assessment of the conservation status of both species within the unique socio-cultural landscapes that they inhabit. In Southeast Asia, the growing value of wild meat and its constituents have provided strong incentives for the illicit trade of wildlife and the unsustainable hunting or poaching that drives it^3^. Wild felids are particularly vulnerable to trade activities, as they tend to live at low population densities and can command a high price in the illegal and legal global markets for their tacit cultural importance as whole skins and skulls^14,15^. While tigers (*Panthera* *tigris)* are thought to dominate the illegal trade of felids in Southeast Asia^16^, local extirpations of the species are prompting poachers and smugglers to turn to alternatives^15^. These shifts in preferences have already impacted clouded leopards, which represent the highest proportion of illegally traded derivates from Asian big cats in loosely regulated rural border towns in Southeast Asia^17^.

While global wildlife trade is mediated through the Convention on Trade in Endangered Species of Wild Fauna and Flora (CITES), enforcement impinges on the correct labelling of species being traded^18^. The sparse data available on clouded leopards suggest that mainland clouded leopards are under significant threat from wildlife trade and poaching^19,20^. These assessments are based on untested assumptions that animal parts found on the mainland are *N.* *nebulosa,* without accounting for the porous trade borders that exists between East and West Malaysia, where both species exist. The absence of simple methods to rapidly distinguish between mainland clouded leopards and Sunda clouded leopards makes it difficult to test the veracity of species labels as they appear on the market. Multivariate skull measurements were effective in distinguishing the two species^21^ due to large differences pertaining to skull, mandible and dentition. However, this study relied on measurements from 136 computed ratio metrics, which are difficult to measure in situ (e.g., wildlife markets). A simplified method that relies on only a handful of highly explanatory skull variables would enable rapid identification of the species in a field-based setting, where sophisticated tools may not be readily available^22^.

Furthermore, the relatively recent taxonomic reclassification of clouded leopards introduces an important challenge of correctly re-labelling specimens collected prior to the change, exacerbated by the lack of information on geographical origin for a substantial portion of the specimens available in these collections (Yamaguchi unpublished). Unprovenanced specimens are usually labelled as *Neofelis* spp., which renders them problematic and unavailable to morphological and molecular research concerning *N.* *nebulosa* and *N.* *diardi.* Much of the taxonomic confusion concerning *Neofelis* spp*.* was caused by nearly two centuries of uncertainty about the origin of the type specimen first described by Griffith in 1821^21^. Natural history collections are a crucial point of reference for exploring evolutionary patterns in biology in a systematic framework. If unprovenanced specimens of *Neofelis* spp*.* could be assigned to either *N.* *nebulosa* or *N.* *diardi* both easily and reliably, it would surely increase the scientific value of these collections.

This study sets out to test a statistical pipeline for identifying simple morphological skull measurements and non-metric skull characters to distinguish between the two clouded leopard species with a specific focus on easy-to-use field-based applications (including museum collections). We aim to achieve this by (1) identifying skull morphological variables that have the greatest discriminatory power to distinguish between Sunda and mainland clouded leopards, (2) fitting and testing the accuracy of predictive models for species identification and (3) developing a web-based tool for calculating likelihood of species identity based on skull measurements.

## Results

### Categorical features: fronto-nasal “pit” and m_1_ talonid

**Fig. 1:**
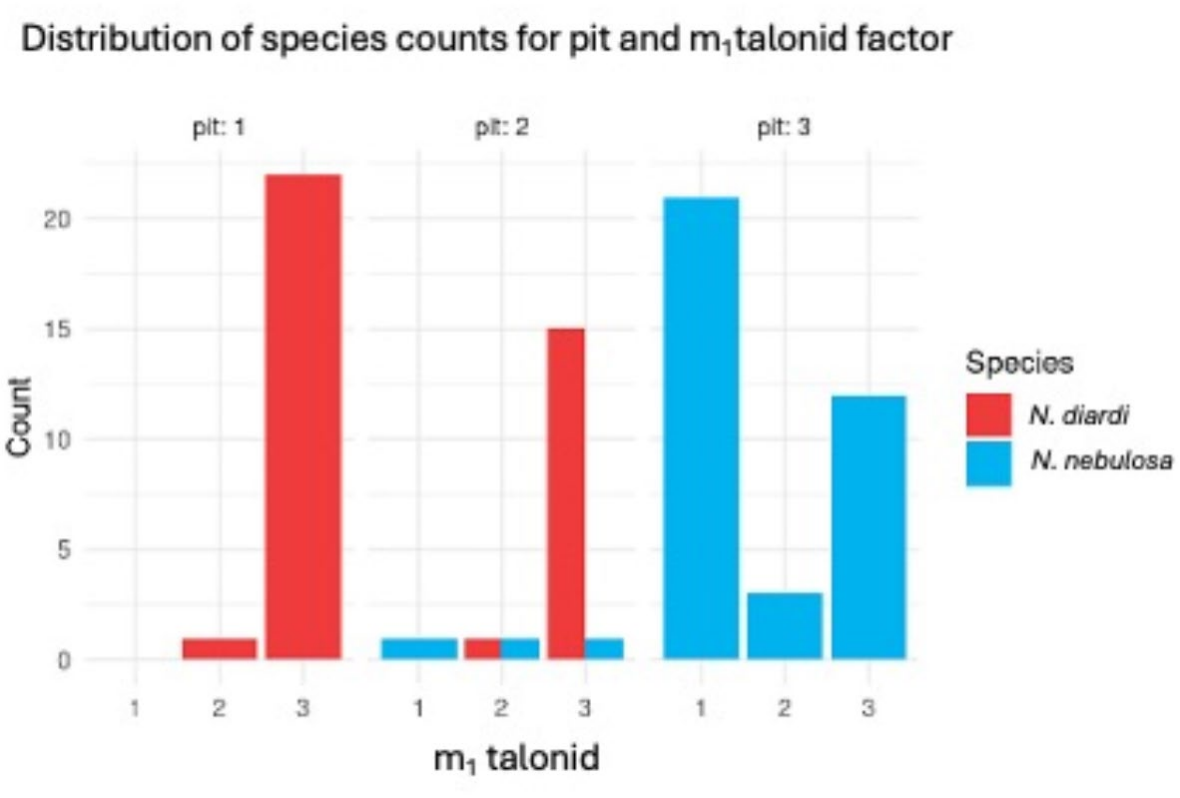
Frequency distribution of the fronto-nasal pit scores against m_1_ talonid scores of N. *nebulosa* and *N.* *diardi* skulls.

There was strong evidence that the degrees of developments of the fronto-nasal “pit” and lower first molar (m_1_) talonid varied between *N.* *nebulosa* and *N.* *diardi* where ‘pit’ (Chi squared test: *df* = 2, likelihood ratio χ^2^ = 94.28, *p* < 0.001) and m_1_ talonid (Chi squared test: *df* = 2, likelihood ratio χ^2^ = 39.19, *p* < 0.001). The frequency distribution of a combination of the two categorical variables on the same skull is summarised in Fig. 1. The logistic regression model built using both predictors recorded an AIC and AUC scores of 22.57 and 0.9842 respectively (Table 3). The model accuracy tested, using a confusion matrix, was 0.9615 (*p* < 0.001) with sensitivity and specificity of model at 1.0 and 0.949 respectively.

### Age and sexual dimorphism

**Fig. 2:**
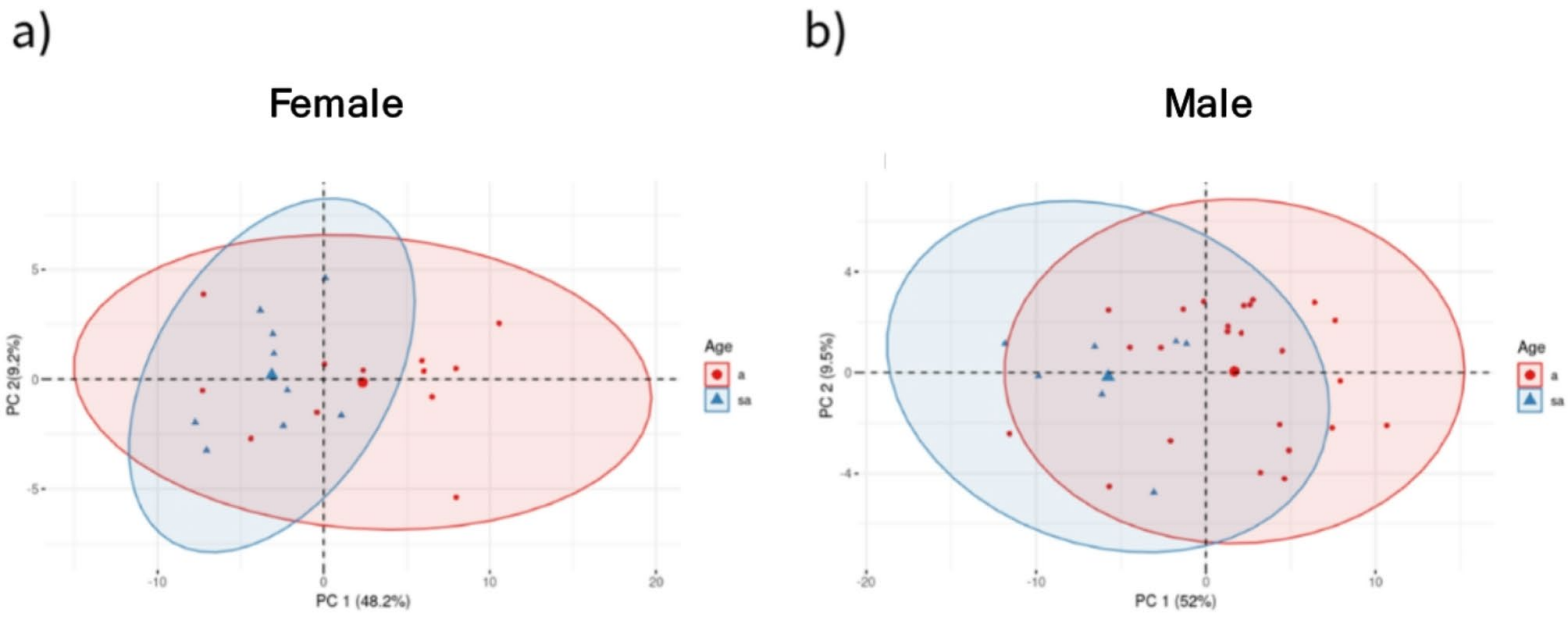
PCA plots showing overlap in skull shapes of the adult and subadult age-classes for both female(**a**) and male (**b**) clouded leopards, *Neofelis* spp.

To test the difference between adults and subadults, we used the full dataset available for males (N_a_ = 24, N_sa_ = 7) and females (N_a_ = 12, N_sa_ = 9). After removing variables that had a skewed distribution of values (> 0.5) and missing values, we retained 67 continuous scaled variables. PC1 and PC2 were plotted separately for males and females and showed substantial overlap between measurements for adults and subadults (Fig. 2a and b), allowing us to pool subadult and adult specimens for both species without confounding species discrimination signals with age effects.

**Fig. 3:**
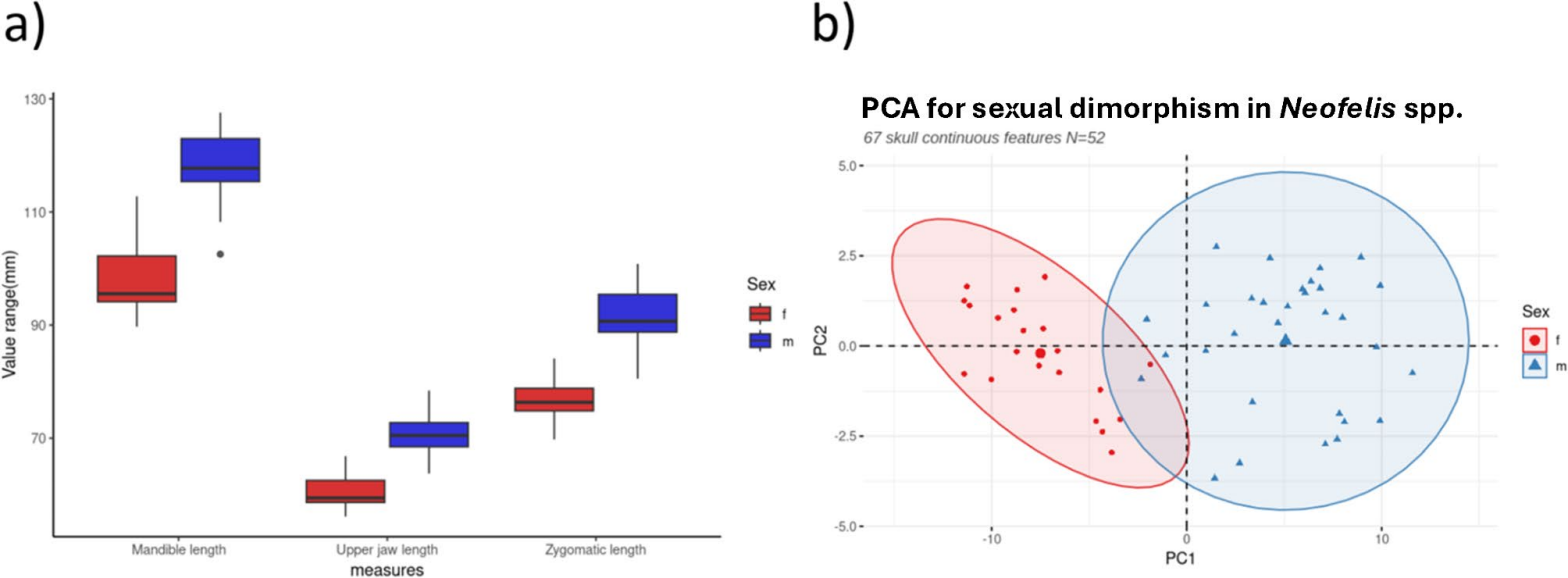
(**a**) PCA plot visualising the dimorphism in continuous measurements of the skull between males and females of *Neofelis* spp. Loadings from PC1 were used to determine variables that contribute to highest variation in the dataset, which were subjected to t-tests. (**b**) Boxplot showing the size-range differences between males and females for the three selected measurements.

PC1 and PC2 of the combined PCA were able to explain 82% of total variation between the two sex clusters (Fig. 3b). The non-model PCA clusters were clearly separated using the complete 67 continuous variables. From a rank list of the 10 features with the highest loadings in PC1, we selected zygomatic length, mandible length and upper jaw length for their ease of measurement. A boxplot clearly shows near complete separation in size ranges for the chosen skull variables between males and females (Fig. 3a). Welch’s t-test with Bonferroni correction yielded a significant result for all three chosen measurements (Table 1).

**Table 1:** T-tests for three variables selected from the first principal component as having the highest variation between males and females.

Measures	n1	n2	t-statistic	df	*p* value
Zygomatic length	20	32	− 14.05	48.98	8.36E−19
Mandible length	20	32	− 12.3	41.31	2.13E−15
Upper jaw length	20	32	− 11.26	45.14	1.07E−14

### Species differentiation: continuous features

A total of 31 males (N_*nebulosa*_ = 12, N_*diardi*_ = 19) were used to fit a predictive model from the 67 continuous features. After removing variables that were difficult to measure, correlated ( >|0.6|) or with a high variance inflation factor (> 0.3), the final global male logistics regression model was constructed using five continuous predictors. The top model with ΔAICc = 0 was a univariate model with postorbital breadth as a significant predictor (Table 2). The null model had a ΔAICc value > 2, indicating significant support for top model predictor performing better than the null model. When evaluated using the most suitable threshold of 0.45, the top model performed significantly well. (*p* < 0.05) (Table3) with an overall prediction accuracy of 0.81 (95% CI0.63, 0.93), sensitivity (0.84), specificity (0.75) and an AUC value of 0.77 (Fig. S3).

**Table 2:** Test statistics for top continuous model for postorbital breadth of *Neofelis* spp. postorbital breadth as ranked by AICc for males and females separately (* for p<0.05).

Model	Predictor	Coefficient	Standard error	*P* value
Male	Intercept	18.356	7.8247	0.019*
	Postorbital breadth	− 0.634	0.2640	0.016*
Female	Intercept	43.132	20.4690	0.035*
	Postorbital breadth	− 1.551	0.7338	0.034*

Model outputs were used in web-based app to calculate likelihood of species identity.

**Table 3:** Confusion matrix results from predictions made for three GLM models constructed using categorical predictors, male continuous variables and female continuous variables for skulls of *Neofelis* spp.

Model	Model performance indicators				
	Accuracy	*p* value (Acc > NIR)	Sensitivity	Specificity	AUC
Categorical predictors	97%	< 2e−16	1	0.945	0.99
Male continuous model	80.6%	0.018	0.842	0.75	0.77
Female continuous model	85.7%	0.017	0.769	1	0.87

The female model, using continuous features, was constructed using data from 21 individuals (N_*nebulosa*_ = 13, N_*diardi*_ = 8). After removing correlated predictors, the global model was constructed using five predictors. Full subset analysis of the global model identified a best model with only postorbital breadth as the best model (Table 2). The null model had a ΔAICc value > 2 indicating significant support for the highest-ranking models and thus rejecting the null hypothesis. The model performed best using a threshold of 0.41. Predictions were significant (*p* < 0.05) (Table 3) with an overall accuracy of 0.86 (95% CI 0.64, 0.97) for classifying species. Sensitivity and specificity were 0.77 and 1.0 respectively, with an AUC value of 0.87 (Fig. S3).

## Discussion

In this paper, we assessed the feasibility of using simple continuous and categorical measures to distinguish between two morphologically similar species. Our results indicate that predictive models constructed using data from museum specimens can perform well, reinforcing the importance of safe storage and accurate labelling of biological specimens in natural history museums. Our results also demonstrate the effectiveness of using craniometric variables for resolving the differences between the two clouded leopard species. When controlling for sex, we found strong overlap in the morphospace of adults and subadults, allowing us to pool subadults with adults into our analysis.

Our results are consistent with previous research^21^ which detected strong sexual dimorphism in *Neofelis,* with several measurements having little overlap in size between males and females. Data from live-trapped Sunda clouded leopards show that several male measurements were nearly twice as large for several recorded skull measures^23^. The high degree of sexual dimorphism of clouded leopards is consistent with current theories that link sexual dimorphism in carnivorans with uni-male breeding systems, especially where functional demands of diet are different between sexes^24^. Our results provide putative evidence that clouded leopards may be amongst the most dimorphic of the extant felids, with dimorphism comparable to that of leopards and lions, which are generally considered to be the most dimorphic extant felids^25^.

**Fig. 4:**
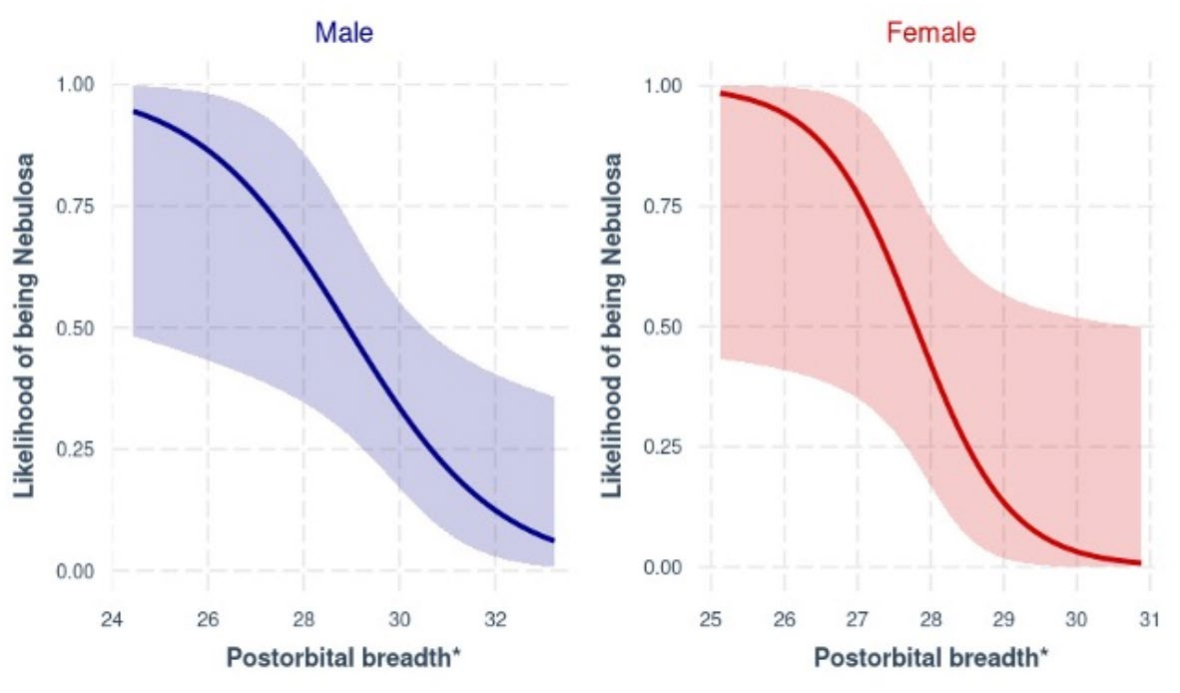
Effect plot of postorbital breadth from male and female logistics regression model with species as a binary response (with 95% confidence interval). A value of 1 on y-axis corresponds to the likelihood of being *N.* *nebulosa.*

We were able to statistically identify a single continuous measure for reliably distinguishing between both species (Fig. 4). Postorbital width is both easy and reliable to measure in situ (Fig. S4)*.* Our results are consistent with results from Christiansen et al.^21^, who also found significantly greater postorbital widths in Sunda clouded leopards compared to mainland clouded leopards. Previous analyses were performed using multivariate statistics such as principal component analysis to identify features that are explanatory for species distinction. The added benefit of our modelling pipeline is firstly based on direct measurements that are easy to measure, and secondly, the ability to model predictions given a new dataset, as demonstrated in the resulting web-based application. The categorical measures of the fronto-nasal “pit” and m_1_ talonid have the added benefit of requiring only visual inspection. With good reference images investigators would be able to also distinguish species from photographs that circulate online, if the skulls are photographed in such a way that these characters can be seen. Online platforms have become a vehicle for illicit trade in countries where physical wildlife markets are not accessible^26^.

The predictive models generated in this paper offer a pragmatic and replicable framework for creating a quick and inexpensive method for identifying species from morphometric data. The narrow ecological niche and conserved craniometric ontogeny of the Felidae^27^ explain the limited phenotypic radiation within the *Neofelis* genus. This renders them easy targets of intentional and unintentional mislabeling in the context of global trade. The case of *Neofelis* spp. illustrates simultaneously the difficulty and importance of having tools to diagnose rapidly species from cranial data. Current trade dynamics for *Neofelis* spp. do not identify the island range of *N.* *diardi* as a potential source for the trade of *Neofelis* spp. in continental Asia^19^. However, these models do not consider the porous borders that exist between the Sunda islands of Borneo and Sumatra with mainland Asia, which have shared national borders and/or have poorly regulated informal borders.

A large portion (~ 30%) of the specimens evaluated in this study did not have data on provenance, rendering them unusable for training our model. Therefore, this tool will also enable the assignment of provenance to specimens of *Neofelis* *spp.* in natural history collections around the world. This tool will also enable museum curators to expand the relevance and utility of these unprovenanced specimens in research and education.

We used all three of our best models, which include the two categorical predictors (“pit” and m_1_ talonid) and the uni-variate continuous models for males and females. We built separate models of continuous craniometric variation for each sex because of the significant variation in measurements due to sexual dimorphism^28^. The models fitted with continuous measurements were weaker compared to the logistic regression model using the fronto-nasal pit and m_1_ talonid categorical variables. Continuous cranial measurements vary in relation to skull shape and size. For species with limited morphological variation, discrete trait data can often perform better at parsing cryptic variation^29,30^. The use of a few morphological measurements negates the need for specialised training and specific tools that can add constraints to their application for regulators and enforcement agencies. Research in the use of DNA sequencing technology has been useful for cracking down on the trade of wildlife derivates^31^ is technologically prohibitive in remote areas and for institutions with limited funding^32^. Samples for DNA analysis may also have to be sent to labs outside range countries and CITES legislation makes this almost impossible or too slow for practical purposes.

Our web-based tool will allow users to input new data to probabilistically determine species identity for the *Neofelis* genus using cranial measurements. The tool estimates the likelihood of skulls belonging to either species by fitting data into the model using variable coefficients generated when training the model. Our model coded into the app will now allow practitioners to quickly identify and calculate the probability of species identity for *Neofelis* spp. This paper also provides a practical framework for creating more usable applications for species identification to increase access to practitioners of scientific models and tools. The web-application for *Neofelis* species prediction tool can be found using the following link: https://wildcru-oxford.shinyapps.io/test_code/. Free and usable tools like this will leverage the wealth of resources stored in natural history collections for solving the most pressing challenges in wildlife conservation.

## Methods

### Data collection

Skulls of clouded leopards were examined for 11 potential skull characteristics (Table S1) for distinguishing between the two clouded leopard species. We identified two characters: the depth of the fronto-nasal “pit” (scored either 1, 2 or 3: Fig. S1) and the shape of the talonid of first lower molar (m1) (scored either 1, 2 or 3: Fig. S2), as potentially useful. An additional 75 morphometric variables per skull were also collected from museum collections in Europe, North America and Asia following Cooper et al.^33^. All measurements were made by the same examiner (NY), minimising any variation introduced by examiner-related variation. We present here the largest collection of skull measures for *Neofelis* spp. collected to date. We have examined 122 skulls in total consisting of 43 *N.* *nebulosa*, 42 *N.* *diardi*, and 37 *Neofelis* spp. (of unknown origin). Only 52 skulls out of the total dataset had less than 10% of missing data and were used in training the final classification model (32 *N.* *diardi* and 20 *N.* *nebulosa*). Provenances were taken from specimen labels and records kept in the collections. The age category (adult, subadult, or juvenile) of an animal was determined by following Yamaguchi et al.^34^. Specimens were classed as adult if the frontal suture was closed, and subadult if it was open. Juveniles were classed using the visibility of cementoenamel junction of any permanent canine. Sex was taken from museum labels and records if available, and sexual dimorphism of canine size was used to verify it, as well as to determine the sex of any unsexed specimens^24^. Also, we assessed whether an animal was wild, or captive based on museum labels and records.

### Data filtration

Missing data for continuous variables were filled using median imputation, which is favoured over mean, because it does not distort the shape of the distribution of values and means for a given variable^35^. To explore the treatment of the “subadult” and “adult” age categories, we performed a principal component analysis using the continuous variables to test differences between the age groups within each sex category. We also removed variables, which would be difficult or unreliable to measure in situ. We defined difficult or unreliable as any measurement that would (1) require a specialised measuring instrument, (2) is not visible to untrained personnel and (3) cannot be easily accessed.

### Data analysis

We identified the most meaningful predictors for sex differentiation by calculating the principal components of the scaled continuous variables. We did this by comparing the loadings on the first principal component to identify variables with the most variation in the dataset. We then performed a t-test to test the null hypothesis that these scores did not differ between the sexes.

To classify species using categorical predictors, we used a contingency table Chi-squared test to detect whether there were statistically significant differences in the prevalence of the fronto-nasal “pit” or m_1_ talonid between *N.* *nebulosa* and *N.* *diardi.* We then fitted a logistics regression model with species as a response using the two categorical measures.

After filtering out skewed and missing variables, we scaled the continuous variables and calculated correlations between the remaining variables using the Pearson’s correlation coefficient and removed variables with >|0.6| correlation coefficient.

We fitted a global logistic regression model with the selected variables and calculated the variance inflation factor (VIF) using the *DAAG* package^36^(v 1.25.6). VIF test is an important step when handling collinear ecological and evolutionary data ^37^. We removed all predictors with a high VIF (> 3) and repeated the logistic regression with the remaining predictors. We used the dredge function from the *MuMIn* package^38^ (v 1.48.4) to select the best model as ranked by the Akaike Information Criterion (AICc). A Δ AICc value < 2 for the null model compared with the best performing model will tend to indicate no relationship between response and modelled predictors^39^. We visualised the effect of each predictor in the best model selected for males and females.

We used the *predict* function in *stats* package^40^ (*v* 3.6.2) to test model performance using the same dataset. Partitioning the dataset for training and testing was not appropriate due to the limited number of specimens available. To select the right threshold for species prediction, we used the *PresenceAbsence* package^41^ (*v* *1.1.11*). We evaluated and selected the best threshold for the model using a confusion matrix with statistical metrics for accuracy, specificity and sensitivity of the predicted results. All statistical analyses were carried out in R version 4.3.1^40^.

### App development for in situ application

We developed an R shiny app^42^ to create a graphical user interface for calculating the probability of a set of measurements belonging to either *N.* *nebulosa* or *N.* *diardi*. We used coefficients and variables from our top selected models to build a prediction function by back transforming predictions on the logit scale to probabilities.

## Electronic supplementary material

Below is the link to the electronic supplementary material.

Supplementary Material 1

Supplementary Material 2

## Data Availability

R code used for statistical analysis can be found in supplementary files, and full craniometric data used in this study will be made available by request to corresponding author.
