# Supplementary material for: A web-based tool for rapid and accurate craniometric differentiation of clouded leopard species
Source: Sci Rep. 2025 Apr 2;15:11240. doi: 10.1038/s41598-025-96080-w (PMC11965479; doi:10.1038/s41598-025-96080-w)
Supplement: Supplementary file 2 — Supplementary Material 2 [file 41598_2025_96080_MOESM2_ESM.docx]

**Supplementary Tables and Figures**

**A web-based tool for rapid and accurate craniometric differentiation of clouded leopard species**

Chrishen R. Gomez_­_^1^, Andrew C. Kitchener^2^, Andrew J. Hearn^1^, Ibnu Maryanto^3^, Paul J. Johnson^1^, David W. Macdonald^1^ and Nobuyuki Yamaguchi^1,4^

**1.Wildlife Conservation Research Unit, Department of Biology, University of Oxford,** United Kingdom

**2. Department of Natural Sciences, National Museums Scotland,** United Kingdom and School of Geosciences, University of Edinburgh, United Kingdom

**3. Museum Zoologicum Bogoriense Center for research on biosystematica and evolution-BRIN, Indonesia**

**4. Institute of Tropical Biodiversity and Sustainable Development, University of Malaysia Terengganu,** Malaysia

**Figure S1**

Examples of the forehead “pit”, which was scored either 1 (none or very small), 2, or 3 (very clear) depending on the degree of its distinctness.

a) Score = 1


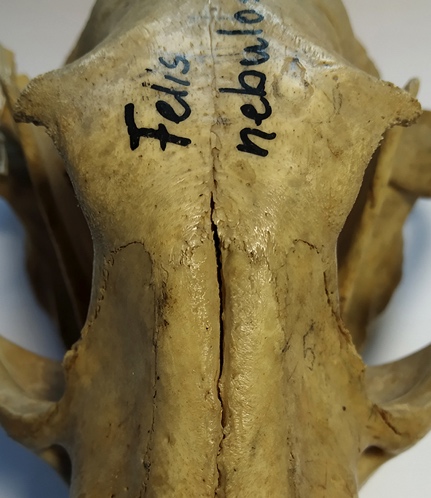


b) Score = 2


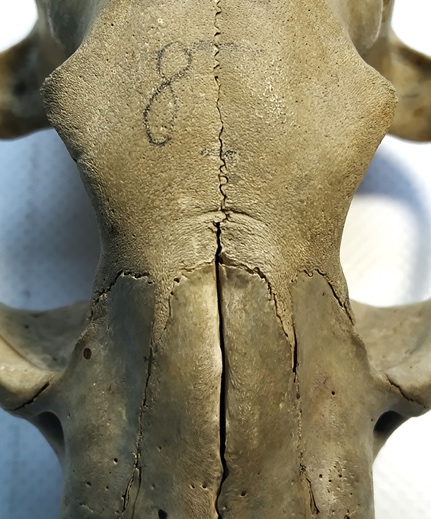


c) Score = 3


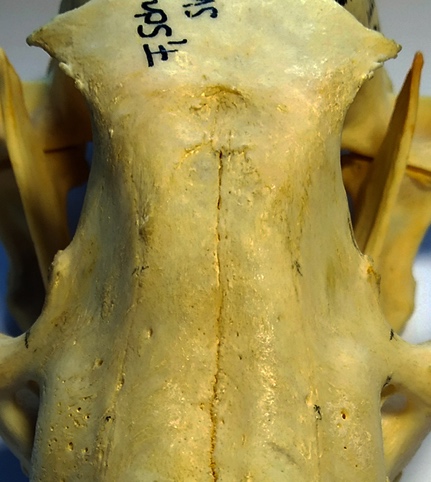


**Figure S2**

Examples of the M_1_ talonid, which was scored either 1 (one big capsule), 2 (one big and one tiny capsule), or 3 (two clearly distinct capsule).

a) Score = 1


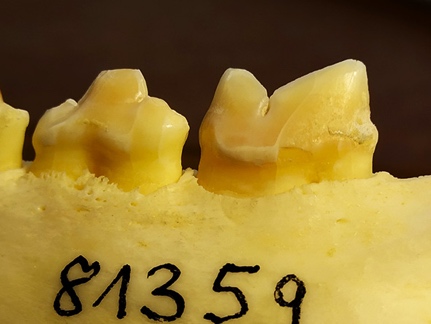


b) Score = 2


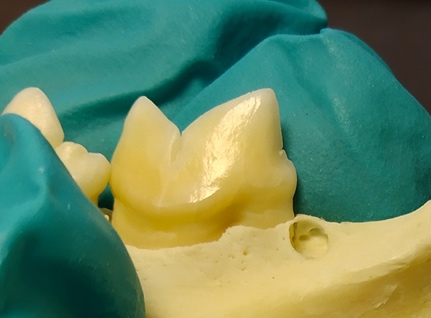


c) Score = 3


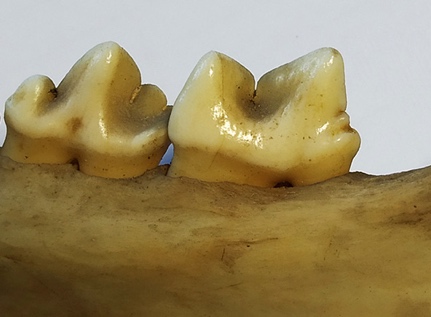


**Figure S3**

AUC curves for predictions from top models of males (a) and females(b).

A) b)


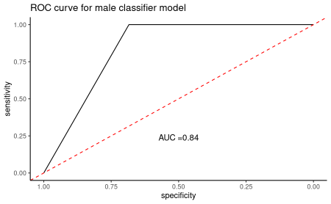

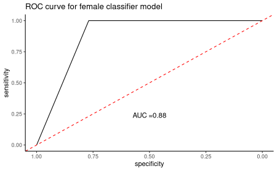


**Figure S4**

Postorbital breadth (labelled 22) feature which can be used to distinguish both male and female clouded leopards using the logistic regression model coded in the application.


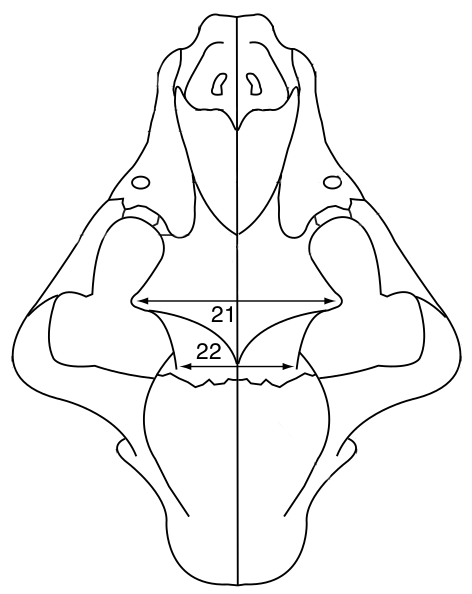


**Table S1**

List of continuous measures and categorical characteristics recorded from each skull for both species.

| Name of Measurement | Type |
| --- | --- |
| Frontal breadth | Continuous |
| Greatest length | Continuous |
| Parlate-inion | Continuous |
| Nasal-inion | Continuous |
| Facial length | Continuous |
| Head length | Continuous |
| Bizygomatic breadth | Continuous |
| Zygomatic length | Continuous |
| Zygomatic length anterior | Continuous |
| Orbit vertical & horizontal | Continuous |
| Postorbital bar | Continuous |
| Facial length | Continuous |
| Saggital crest | Continuous |
| Cranial height I & II | Continuous |
| Cranial height III & IV | Continuous |
| Interorbital breadth | Continuous |
| Postorbital breadth | Continuous |
| Nasal length -I & II | Continuous |
| Nasal breadth | Continuous |
| Breadth between infra orbital formina | Continuous |
| Rostral depth I & II | Continuous |
| Rostral breadth | Continuous |
| Nasal aperture | Continuous |
| Upper jaw | Continuous |
| Palate length | Continuous |
| Palate breadth I & II | Continuous |
| Canine Pm4 & Pm2-Pm4 | Continuous |
| Upper canine height, breadth & breadth-lateral | Continuous |
| Upper canine alveolus: greatest and smallest diameters | Continuous |
| Pm4 length and breadth I & II | Continuous |
| Mastoid breadth | Continuous |
| Skull height I & II | Continuous |
| Foramen magnum breadth & height | Continuous |
| Occipital condyles breadth | Continuous |
| Tympanic bulla length, breadth & breadth | Continuous |
| Mandible length I & II | Continuous |
| Mandible height | Continuous |
| Mandible height I & II | Continuous |
| Maximum width of mandibular condyle | Continuous |
| Mandible depth I &II | Continuous |
| Canine-M1 & Pm3-M1 | Continuous |
| Lower canine height,breadth & breadth-lateral | Continuous |
| Lower canine alveolus: greatest and smallest diameters | Continuous |
| Pm4 length & breadth | Continuous |
| M1 length & breadth | Continuous |
| Lower M1 posterior process | Categorical |
| Forehead pit | Categorical |
| Occipital condyles | Categorical |
| End palate | Categorical |
| Z.A thickness | Categorical |
| Interorbital ramps | Categorical |
| Mandibles rock | Categorical |
| Frontal suture | Categorical |
| Upper Pm2 (L,R) | Categorical |
| Upper M1(L,R) | Categorical |
| IOF (L,R) | Categorical |
| Basal suture | Categorical |
